# Effect of essential oils from *Cymbopogon citratus, Citrus grandis*, and *Mentha arvensis* on *Trichomonas vaginalis* and role of its symbionts *Mycoplasma hominis* and *Ca.* Mycoplasma girerdii

**DOI:** 10.3389/fpara.2025.1610965

**Published:** 2025-08-14

**Authors:** Valentina Margarita, Thi Ha Trinh Nguyen, Giacomo Luigi Petretto, Antonella Congiargiu, Antonietta Ligas, Nicia Diaz, Phuong Anh Ton Nu, Giorgio Pintore, Paola Rappelli

**Affiliations:** ^1^ Department of Biomedical Science, University of Sassari, Sassari, Italy; ^2^ Department of Microbiology and Parasitology, Buon Ma Thuot Medical University, Buon Ma Thuot, Vietnam; ^3^ Department of Medicine, Surgery, and Pharmacy, University of Sassari, Sassari, Italy; ^4^ Department of Parasitology, Hue University of Medicine and Pharmacy, Hue, Vietnam

**Keywords:** *Trichomonas vaginalis*, essential oils, *Mycoplasma hominis*, *Candidatus* Mycoplasma girerdii, *Cymbopogon citratus*, *Citrus grandis*, *Mentha arvensis*, lactobacilli

## Abstract

**Introduction:**

Trichomoniasis, the most common non-viral sexually transmitted disease, is caused by the protozoon *Trichomonas vaginalis*. *T. vaginalis* can establish a symbiosis with two bacteria, *Mycoplasma hominis* and *Candidatus* Mycoplasma girerdii, whose intracellular presence may modulate several characteristics of the protozoan, including its sensitivity to 5-nitroimidazoles, the only class of drugs currently effective in treating trichomoniasis. The rising prevalence of *T.vaginalis* strains resistant to metronidazole, the most commonly used antitrichomonal drug, underscores the need for therapeutic alternatives active against the protozoon.

**Methods:**

In this study, we evaluate the antimicrobial activity of essential oils extracted from three plants cultivated in Vietnam — *Cymbopogon citratus*, *Citrus grandis*, and *Mentha arvensis* — against thirty *T. vaginalis* strains isolated from symptomatic women in Italy and Vietnam. We also assess the influence of *M. hominis* and *Ca*. M. girerdii on *T. vaginalis* susceptibility to essential oils and metronidazole, through dedicated susceptibility assays. Additionally, given the importance of lactobacilli in maintaining vaginal health, we investigate the effects of the essential oils on *Lactobacillus gasseri* and *Lactobacillus crispatus*. The cytotoxic activity of the oils against HeLa cells was also tested *in vitro*.

**Results:**

All three essential oils showed effective antitrichomonal activity without inhibiting lactobacilli growth. Among them, *C. citratus* oil exhibited the strongest inhibitory effect on *T. vaginalis*, including strains harboring bacterial symbionts. Moreover, the oils demonstrated no cytotoxic activity against HeLa cells at the concentrations effective against the protozoan.

**Discussion:**

The results support the potential of *C. citratus* essential oil as a natural antitrichomonal agent. Its effectiveness against both free and symbiont-infected *T. vaginalis* strains positions it as a promising candidate for developing alternative therapies against drug-resistant trichomoniasis.

## 1 Introduction

The protozoan flagellate *Trichomonas vaginalis* is a human parasite responsible for the most common non-viral sexually transmitted disease, with over 150 million new cases reported annually (Wei et al., 2025). In women, the infection can range from asymptomatic to severe vaginitis, characterized by malodorous vaginal discharge, burning, and dyspareunia. In men, trichomoniasis is typically asymptomatic but can be associated with urethritis and prostatitis. *T. vaginalis* infection is also linked to adverse pregnancy outcomes, including an increased risk of low birth weight infants and preterm rupture of membranes (Fichorova, 2009). Moreover, trichomoniasis increases the risk of acquiring HIV and has been linked to a higher likelihood of developing malignant cervical and prostate cancers (Stark et al., 2009; Sutcliffe et al., 2006).

During infection, *T. vaginalis* establishes complex interactions with the vaginal microbiota, altering its composition by reducing the number of protective lactobacilli species (Cardoso and Tasca, 2025). Lactobacilli play a crucial role in maintaining vaginal health by producing lactic acid, which helps sustain an acidic environment that inhibits the growth of pathogenic microorganisms and supports the stability of the vaginal microbiota (Ravel et al., 2011). In the presence of *T. vaginalis*, protective species such as *Lactobacillus gasseri* and *Lactobacillus crispatus* are replaced by anaerobic bacteria associated with vaginosis (Cardoso and Tasca, 2025). It has been shown that *L. gasseri* can reduce *T. vaginalis* adhesion to host vaginal cells, thereby interfering with the protozoan’s cytopathic effect (Phukan et al., 2013).

In 1998, our research group observed that *T. vaginalis* can establish a symbiosis with *Mycoplasma hominis* (Mh), another pathogen found in the human genitourinary tract (Rappelli et al., 1998). *M. hominis* is an obligate parasite belonging to the class Mollicutes, the smallest organisms capable of independent replication. In women, *M. hominis* infection is often asymptomatic but is linked to alterations in the vaginal microbiota and bacterial vaginosis (Rumyantseva et al., 2019). Moreover, *M. hominis* is associated with pregnancy and postpartum complications, including spontaneous abortion, endometritis, and low birth weight (Capoccia et al., 2013). Since its discovery, the close relationship between these two microorganisms, the first known symbiosis between two obligate human pathogens, has been extensively studied. The presence of *M. hominis* has been demonstrated in *T. vaginalis* isolates worldwide, with percentages varying from 5% to 89% (Fichorova et al., 2017), and this association has been shown to influence several aspects of the protozoan’s pathobiology (Dessì et al., 2019).

Interestingly, a second *Mycoplasma* species, *Candidatus* Mycoplasma girerdii (Mg), has recently been described in association with *T. vaginalis*. This new species is found in *T. vaginalis* isolates more frequently than *M. hominis*. *Ca.* M. girerdii exhibits typical *Mollicutes* features and is characterized by a very small genome. Its limited metabolic capabilities justifies its obligate dependence as an endosymbiont of *T. vaginalis*, which provides a protected niche and a source of essential metabolites (Margarita et al., 2022a).

The treatment of trichomoniasis relies on the use of nitroimidazoles, which effectively target the organism’s anaerobic metabolism. Metronidazole (MTZ), a 5-nitroimidazole derivative, has been the standard treatment for trichomoniasis for decades and, along with tinidazole, represents the only recommended drugs for treating *T. vaginalis* infection. Although metronidazole is generally well tolerated, therapy can be associated with adverse effects. Furthermore, metronidazole and other 5-nitroimidazoles are still under scrutiny for their potential carcinogenicity (Leitsch, 2019).

Increasing reports of *T. vaginalis* resistance to metronidazole have raised concerns regarding the long-term efficacy of this drug, and infections refractory to nitroimidazoles are becoming more frequent. Resistance to metronidazole in *T. vaginalis* is a growing issue, highlighting the urgent need for new treatment alternatives (de Brum Vieira et al., 2015). Plant extracts, a major component of various traditional medicines, represent a promising alternative to conventional drugs, given the increasing incidence of antimicrobial resistance (Schwebke and Barrientes, 2006). Essential oils (EOs), in particular, are among the most commonly used plant-derived products, with significant therapeutic potential for treating bacterial, fungal, and protozoan infections. However, the anti-*T. vaginalis* activity of essential oils has been poorly investigated so far (Donadu et al., 2020; Le et al., 2020; Ziaei Hezarjaribi et al., 2019).

In this study, we tested the antiprotozoan activities of three essential oils, widely used in Vietnam for their recognized healing properties, obtained from *Cymbopogon citratus*, *Mentha arvensis*, and *Citrus grandis*. *C. citratus*, commonly called lemongrass or citronella, belongs to the Poaceae family, which includes approximately 500 genera and 8,000 species of plants. *C. citratus* is a perennial plant from Southwest Asia, now grown in all tropical regions of the world. The essential oil extracted from its leaves and stems is widely used in traditional medicine for its antiseptic, anti-inflammatory, and analgesic effects (Kiełtyka-Dadasiewicz et al., 2024). *M. arvensis*, commonly known as corn mint, belongs to the Lamiaceae family and is widely cultivated for its high menthol content (Parić et al., 2024). The essential oil derived from its leaves is prized for its invigorating and cooling properties. It is commonly used in aromatherapy to relieve headaches, improve mental clarity, and reduce stress. Additionally, this oil has antimicrobial and anti-inflammatory effects, making it useful for soothing skin irritations and promoting respiratory health (Parić et al., 2024). *C. grandis*, also called “pomelo”, belongs to the Rutaceae family and originates from Southeast Asia. It is widely cultivated in Vietnam and is used in pharmaceuticals and cosmetics (Anmol et al., 2021).

Studies on *T. vaginalis* isolates resistant to MTZ report percentages varying from 2% to 10%, depending on their geographic origin (Margarita et al., 2022b; Schwebke and Barrientes, 2006). For this reason, we tested the effects of the three essential oils from Vietnam on 30 *T. vaginalis* strains isolated from two different geographical regions, Italy and Vietnam, and correlated the results with the presence/absence of the two *Mycoplasma* symbionts. We have previously investigated the possible correlation between the presence of *Ca.* M. girerdii and/or *M. hominis* in *T. vaginalis* isolates and *in vitro* drug susceptibility, suggesting that the presence of *M. hominis* may increase sensitivity to metronidazole, while the symbiosis with *Ca.* M. girerdii seems to have no effect (Margarita et al., 2022b).

Finally, since an effective anti-*T. vaginalis* drug should selectively target only the protozoon, without affecting lactobacilli to preserve the healthy vaginal microbiota, we tested the activity of the three essential oils against *L. crispatus* and *L. gasseri*, which are key components of the microbiota of the healthy vagina.

## 2 Materials and methods

### 2.1 Gas chromatography/mass spectrometry analysis of essential oils

Three commercial essential oils produced in Vietnam were purchased by Saola (73 Thach Han, Phu Xuan District, Hue City, Vietnam). They were extracted using steam distillation from leaves and stems of lemongrass (*C. citratus*), from cornmint leaves (*M. arvensis*) and from the peel of fresh pomelo (*C. grandis* var. Thanh trà).

The chemical characterization of the essential oil was performed using gas chromatography (GC) coupled with a mass spectrometric (MS) detector. The GC analysis of the essential oils was carried out using an Agilent 6890 GC with an Agilent 7683 autosampler and coupled with an Agilent 5973 MSD detector. The chromatographic separations were performed on a ZB-WAX column 30m ID 0.25mm, 0.25 μm film thickness (Phenomenex).

The following temperature program was used: 40°C hold for 4 min, then increased to 150°C at a rate of 5°C/min, held for 3 min then increased to 240°C at a rate of 10°C/min and finally held for 12 min. Helium was used as the carrier gas at a constant flow of 1 ml/min. The data were analyzed using a Mass Hunter Workstation B.06.00 SP1, and identification of the individual components was performed by comparison against co-injected pure compounds and by matching the MS fragmentation patterns and retention indexes using the built-in libraries, literature data, or commercial mass spectral libraries (NIST/EPA/NIH 2008; HP1607 purchased from Agilent Technologies). A hydrocarbon mixture (linear C8–C23) was injected under the same chromatographic conditions to obtain the linear retention indexes.

### 2.2 Isolation of *T. vaginalis* clinical strains and culture conditions

A total of 30 *T. vaginalis* clinical isolates (hereafter also simply referred to as strains) from women affected by trichomoniasis were included in the present study. The choice to use such a large number of clinical isolates is related to the high phenotypic variability characteristic of *T. vaginalis* (Meade and Carlton, 2013) and to the inclusion of multiple variables in the study, such as geographical origin, MTZ resistance, and the presence or absence of symbionts.

Fifteen isolates were collected by the Laboratory of Microbiology of the University of Sassari (Italy), and 15 isolates by the Department of Parasitology of Hue University of Medicine and Pharmacy (Vietnam). Trichomonad isolates were cultivated by daily passages at 1:16 in Diamond’s TYM (trypticase, yeast extract and maltose) medium supplemented with 10% fetal bovine serum (TYM-FBS) at 37°C in a 5% CO_2_ atmosphere (Clark and Diamond, 2002) for at least 15 days, then protozoa were frozen at −80°C with FBS 90% and 10% dimethyl sulfoxide (DMSO) (Sigma-Aldrich, USA) until use. Only protozoa in an exponential growth phase exhibiting a viability of >95% were used in all experiments.

### 2.3 Screening for *Mycoplasma* endosymbionts in *T. vaginalis* clinical isolates

The presence of *M. hominis* and *Ca.* M. girerdii was assessed in all 30 *T. vaginalis* isolated by extraction of genomic DNA with DNeasy Blood & Tissue Kit (Qiagen Ltd., West Sussex, UK) according to the manufacturer’s protocols. Then, the presence of *M. hominis* and/or *Ca.* M. girerdii in association with each *T. vaginalis* strain was assessed by real-time PCR (qPCR) using the CFX96 Touch real-time thermal cycler (Bio-Rad, Hercules, CA) as previously described. In detail, a primer set that amplifies a fully conserved gene fragment coding for the surface lipoprotein MHO_0730 was used to detect *M. hominis* while *Ca.* M. girerdii was identified by primers specific for the full-length 16S rRNA gene (Cacciotto et al., 2019; Margarita et al., 2022a).

### 2.4 Metronidazole susceptibility assay

All 30 *T. vaginalis* clinical isolates included in the present study were examined for their susceptibility to metronidazole under aerobic conditions. Exponentially growing *T.vaginalis* were centrifuged at 1500 rpm for 10 min and resuspended in TYM-FBS medium at a concentration of 1 × 10^4^ cells/ml. Cells were then exposed to increasing concentrations of metronidazole (range 0.2 to 200 µg/ml) in 96-well flat-bottomed microtiter plates. After 48 h of incubation in 5% CO_2_, microscopic observation was used to determine the minimal lethal concentration (MLC), defined as the lowest drug concentration at which no living trichomonads were detected. Each experiment was conducted twice, with triplicate samples for each isolate. Untreated cultures of each strain served as controls. Strains with an MLC ≤ 25 µg/ml were classified as metronidazole sensitive, while an MLC of 17.1 µg/ml indicated a reduced sensitivity to the drug.

### 2.5 Anti *Trichomonas vaginalis* activity test of EOs

All *T. vaginalis* strains were tested for their susceptibility to *C. citratus*, *M. arvensis* and *C. grandis* EOs. Briefly, exponential growth phase protozoan cultures (viability >95%), were centrifuged at 1500 rpm for 10 min and cells were resuspended in TYM-FBS at a concentration of 1 × 10^4^ cells/ml. Stock solutions were prepared with 2% of each EO plus 8% of DMSO in TYM-FBS medium, then serially diluted in 100 µl of the same medium in 96-well plates. 100 µl of the *T. vaginalis* suspension was added to each well reaching final EO concentrations ranging from 1% to 0.008% (*v/v*) corresponding to a range of 8 mg/ml to 60 µg/ml. The same dilutions were obtained from a stock solution containing 8% of DMSO without EO to be used as a control. Culture plates were incubated at 37°C in a 5% CO_2_ atmosphere and protozoa viability was evaluated after 24 h and 48 h by microscopic observation. The MLC, defined as the lowest essential oils concentration that reduces the viability of the initial microbial inoculum by ≥99.9%, was evaluated.

Each experiment was conducted twice, with triplicate samples for each isolate.

### 2.6 Cytotoxicity assay

Human cervix epithelioid carcinoma cell lines (HeLa) were cultured in RPMI 1640 medium (Gibco™ — USA) supplemented with 10% of FBS, penicillin (100 units/ml) and streptomycin (100 μg/ml) at 37°C in a humidified atmosphere of 5% CO_2_. HeLa were used in the following experiments at ~80% confluency. HeLa cells were seeded at 2 x 10^4^ cells/well in 96-well microplates in RPMI medium plus 10% FBS and allowed to adhere overnight. Stock solutions prepared with 2% of each EO plus 8% of DMSO in RPMI+FBS medium were serially diluted in 200 µl of the same medium (range 1% to 0.008% *v/v*). The supernatants of HeLa cells in microwell plates were then replaced by 200 µl of the diluted essential oils and cells were incubated at 37°C in a humidified atmosphere of 5% CO_2_ for 24 h. The same dilutions were obtained from a stock solution of RPMI containing 8% DMSO without EO to be used as a positive control. Cell proliferation was evaluated by MTT (3-(4,5-dimethylthiazol-2-yl)-2,5-diphenyltetrazolium bromide) assay as previously described (Mosmann, 1983). Cell viability was detected by measuring the optical density at 570 nm and expressed as a percentage of cell viability compared to the positive control.

Each experiment was performed in duplicate and at least two times.

### 2.7 Activity of EOs against lactobacilli

Three clinical isolates of *L. gasseri* and three of *L. crispatus* were collected at the Microbiology Laboratory of the University of Sassari and grown in De Man, Rogosa and Sharpe broth (MRS) at 37°C in anaerobic conditions, using anaerobic atmosphere generating bags (Thermo Fisher Scientific, Rodano, Italy).

The antibacterial activity of *C. citratus, C. grandis*, and *M. arvensis* was evaluated on each clinical isolate of *L. gasseri* and *L. crispatus.* Briefly, stock solutions were prepared with 2% of each EO plus 8% of DMSO in MRS medium, then serially diluted in 100 µl of the same medium (range 1% to 0.008% *v/v*) in 96-well plates. For each bacterial isolate a suspension in MRS broth was prepared with a final concentration of 2x 10^5^/ml and 100 μl were added to the oil dilutions. The same dilutions were obtained from a stock solution of MRS medium containing 8% DMSO without EO and were used as a control. The 96-well plates were incubated at 37 °C for 24 h in anaerobic conditions. The minimum inhibitory concentration (MIC), defined as the lowest drug concentration that inhibits the visible growth of a microorganism at the end of the incubation period (Andrews, 2001), was detected by the naked eye. The sample well with the lowest concentration judged clear was identified as the MIC. A definite turbidity was necessary for an acceptable positive control well.

### 2.8 Selective index

The selective index (SI), also known as therapeutic index, is defined as the ratio between the IC_50_ value of *C. citratus, C. grandis* and *M. arvensis* obtained for HeLa cells and the MLC median value found for *T. vaginalis* strains. The essential oil could be assumed as bioactive and non-toxic with SI> 1. A SI<1 means that the essential oil could be toxic and cannot be used as medical treatment (Indrayanto et al., 2021).

The SI was calculated using the following equation:


$$\text{SI}=\frac{IC_{50}\;\text{(}\text{HeLa cells}\text{)}}{\text{MLC }T.\;vaginalis\;\left(\text{median value}\right)}$$

### 2.9 Statistical analysis

Statistical analysis and graphs were performed using Prism v9 (GraphPad, San Diego, CA). The distribution of samples was assessed using the Kolmogorov–Smirnov and Shapiro tests. Analysis of variance using one-way ANOVA and Mann–Whitney U test, was performed to evaluate significant differences among groups. A p-value <0.05 was considered statistically significant.

## 3 Results

### 3.1 Chemical characterization of essential oils

The chemical composition of all three essential oils was determined by GC-MS analysis and the results were reported in **Supplementary Tables S1**–**S3**.

**Figure 1:**
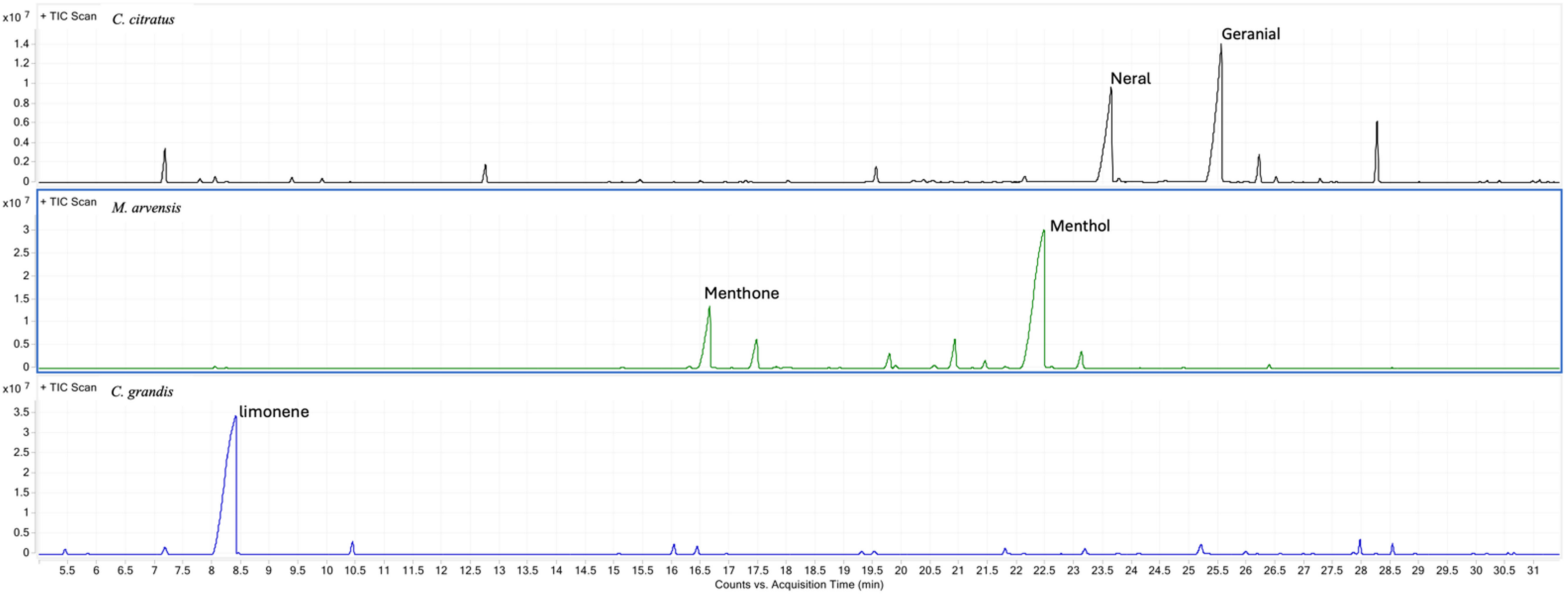
Chemical composition of all three essential oils determined by GC-MS analysis. Typical GC/MS chromatograms of the *C. citratus, M. arvensis* and *C. grandis* essential oil with the identification of the main compounds.

In particular, a total of 30 compounds were identified in *C. citratus* EO, mainly characterized by a high content of the two isomers, neral and geranial, which together accounted for over 73% of the total composition. For *M. arvensis*, 18 active components were identified, with menthol being the most abundant (67.2%), followed by menthone (14.4%), while the remaining active compounds made up 18.4% In *C. grandis*, 25 compounds were detected, with a predominance of limonene which accounted for over 82% of the total composition (**Figure 1**).

### 3.2 *Mycoplasma hominis* and *Ca.* Mycoplasma girerdii detection in *T. vaginalis* clinical strains

In this study, qPCR screening was performed on 15 *T. vaginalis* strains isolated in Vietnam and 15 in Italy to detect the presence of the two symbionts *M. hominis* and *Ca.* M. girerdii. *M. hominis* was detected in 26% of the Vietnamese isolates and 66% of the Italian isolates, while *Ca.* M. girerdii was present in 26% and 40% of the strains from Vietnam and Italy, respectively. Notably, a substantial co-occurrence of both *Mycoplasma* species was observed among the Italian isolates (33%) and Vietnamese isolates (13%). Additionally, 61% of the Vietnamese strains were completely free of *Mycoplasma*, whereas this percentage was significantly lower (27%) among the Italian strains (**Table 1**).

**Table 1:** Prevalence of *M. hominis* and *Ca.* M. girerdii in *T. vaginalis* isolates from Vietnam and Italy.

Microbial Association	*T. vaginalis* (Vietnam) N (%)	*T. vaginalis* (Italy) N (%)
*T. vaginalis* Mh^neg^ Mg^neg^	9 (61%)	4 (27%)
*T. vaginalis* Mh^pos^ Mg^neg^	2 (13%)	5 (33%)
*T. vaginalis* Mh^neg^ Mg^pos^	2 (13%)	1 (7%)
*T. vaginalis* Mh^pos^ Mg^pos^	2 (13%)	5 (33%)

### 3.3 Metronidazole susceptibility of *T. vaginalis* and the symbiosis with mycoplasmas

All 30 *T. vaginalis* isolates were evaluated for their *in vitro* susceptibility to metronidazole (MTZ). The MLC was determined under aerobic conditions following 48 h of incubation at 37°C. The overall mean MLC was 4.5 µg/ml, with Vietnamese strains exhibiting a slightly higher average (5.0 ± 4.3 µg/ml) compared to Italian strains (4 ± 4.3 µg/ml). A MLC of 17.1 µg/ml, indicating a reduced susceptibility to metronidazole, was observed in two isolates, one from Italy (SS22) and one from Vietnam (Tv129) (**Table 2**).

**Table 2:** Metronidazole MLC value in Vietnamese and Italian *T. vaginalis* strains after 48 h of treatment.

MLC (µg/ml)	*T. vaginalis T*otal N (%)	*T. vaginalis* (Vietnam) N (%)	*T. vaginalis* (Italy) N (%)
¾ 4.3 µg/ml	21 (70%)	10 (67%)	11 (73%)
8.6 µg/ml	7 (23%)	4 (26%)	3 (20%)
≥ 17 µg/ml	2 (7%)	1 (7%)	1 (7%)

The antimicrobial susceptibility of 30 trichomonad strains was evaluated *in vitro*, leading to minimal lethal concentration.

**Figure 2:**
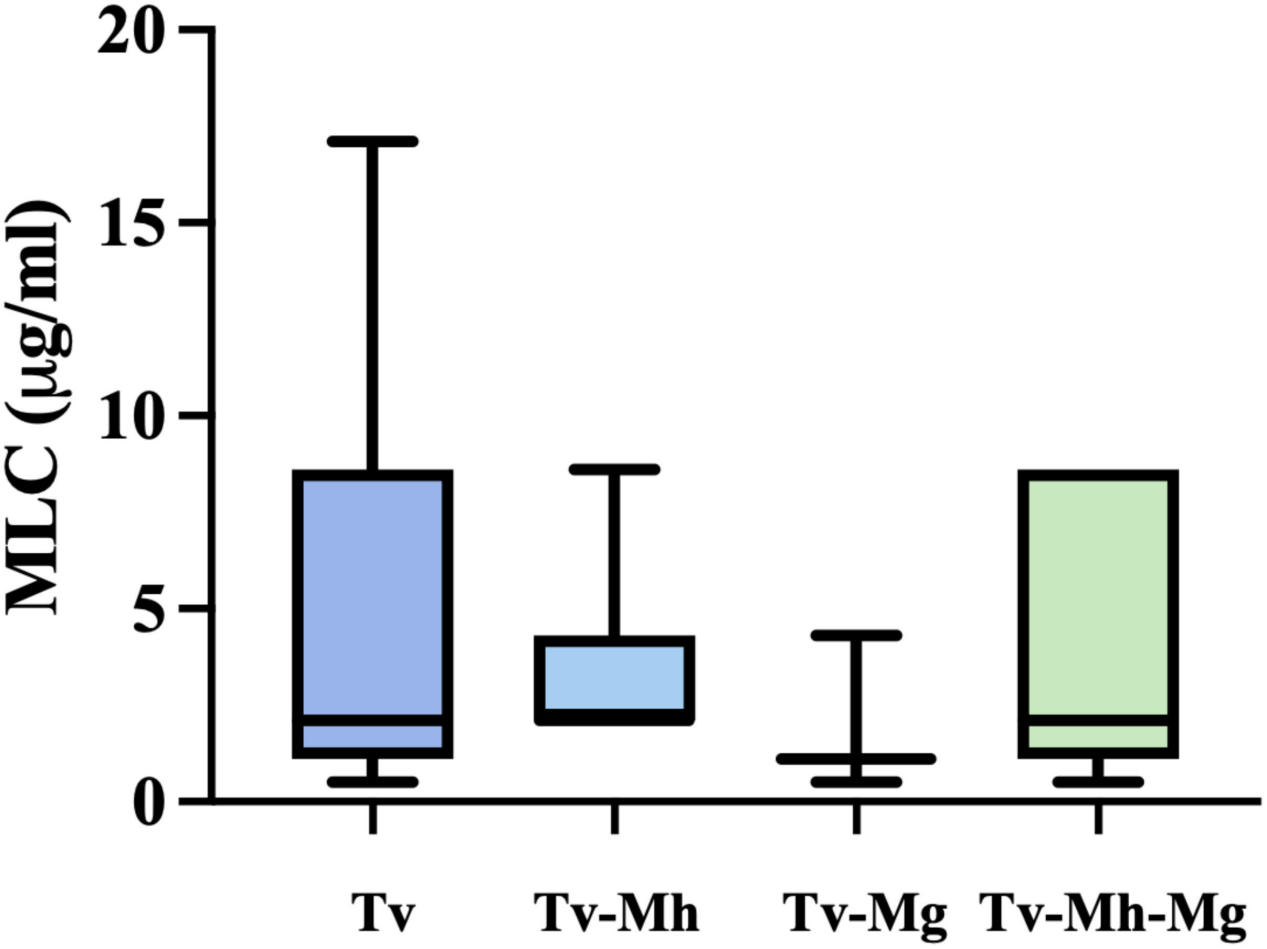
Influence of endosymbionts presence on sensibility of metronidazole of *T. vaginalis* isolates. Tv = *T. vaginalis*, Mh = *M. hominis*, Mg = *Ca.* M. girerdii. The presence of *M. hominis* and *Ca.* M. girerdii in *T. vaginalis* clinical isolates in Vietnam and Italy seem not to impact the sensitivity on MTZ. The horizontal line within each box indicates the median, while the box boundaries represent the first and third quartiles. The vertical lines (whiskers) extend to the minimum and maximum values obtained from a minimum of 3 samples. Statistical significance was tested via the Mann-Whitney test.

Moreover, the association between the presence of *M. hominis* and/or *Ca.* M. girerdii and the sensitivity to metronidazole in *T. vaginalis* isolates were evaluated. As shown in **Figure 2**, there are no statistically significant differences in the mean MLC values among protozoa *Mycoplasma*-free and *T.vaginalis* strains associated with one or both *Mycoplasma* symbionts. Interestingly, *T. vaginalis* strains TV129 (Vietnam) and TvSS22 (Italy) showing a reduced sensitivity to MTZ (MLC value ≥ 17 µg/ml) are totally *Mycoplasma-*free.

### 3.4 Essential oils susceptibility of *T. vaginalis* strains and the symbiosis with mycoplasmas

**Figure 3:**
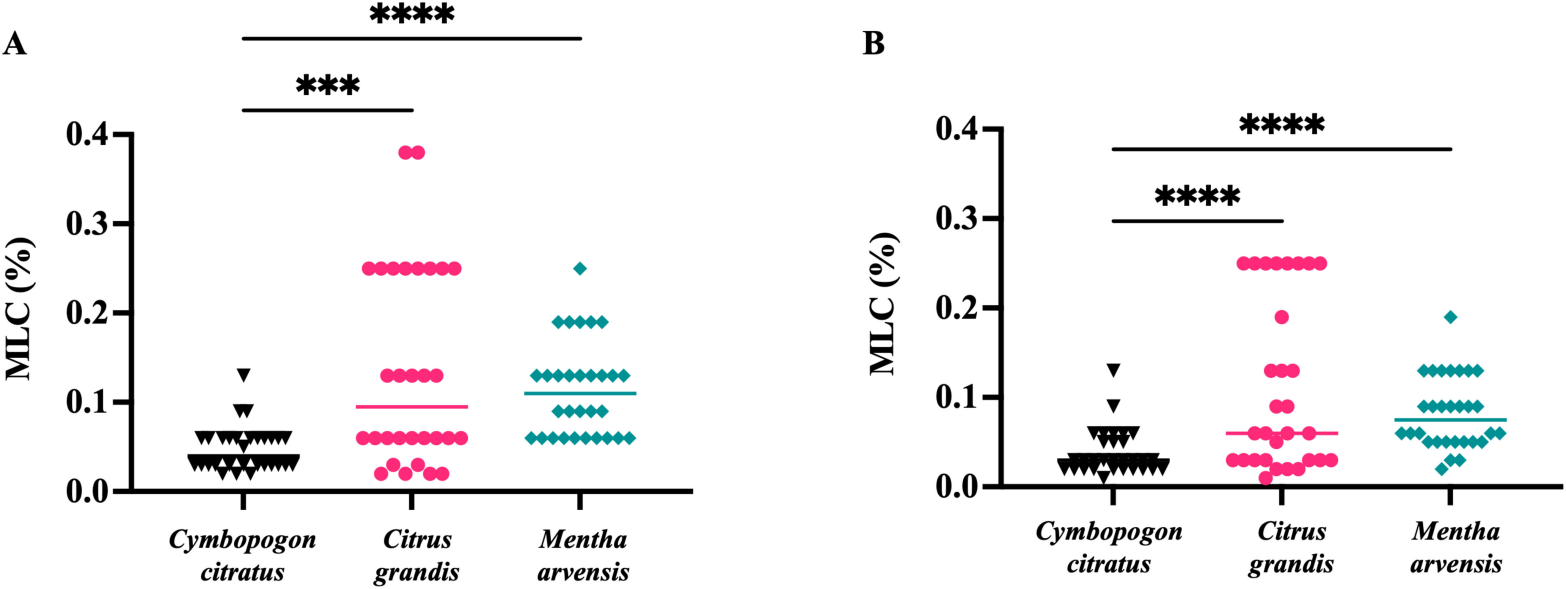
*In vitro* antitrichomonal activity of three essential oils. The inhibitory effects of *C. citratus*, *C. grandis* and *M. arvensis* on *T. vaginalis* isolates growth in different concentration and incubation time. Values are represented as single dot and the bars represent median. **(A)** *C. citratus* showed higher antiprotozoal activity than *C. citrus* (p<0.001) and *M. arvensis* (p<0.0001) at 24 h. **(B)** The higher antitrichomonal activity of C. citratus compared with *C. citrus* (p<0.0001) and *M. arvensis* (p<0.001) was further confirmed at 48 h. Statistical analysis was performed using Friedman test. ***: p<0.001; ****: p<0.0001.

**Figure 4:**
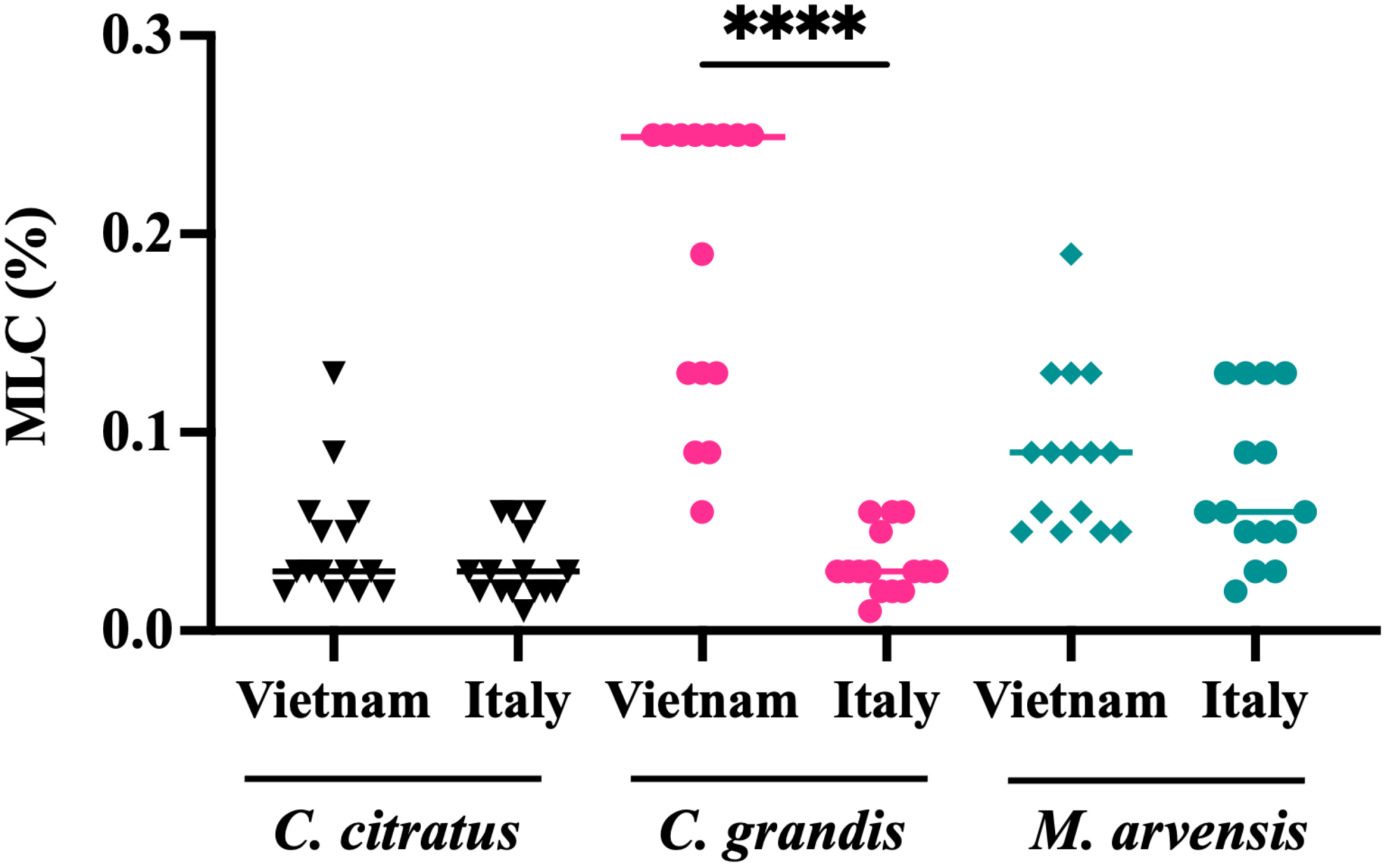
Dependence of the susceptibility of *T. vaginalis* strains according to geographic origin. After 48h, the antitrichomonal effects of *C. grandis* were stronger on Italian *T. vaginalis* isolates than on Vietnamese strains, with a p<0.0001 (****). Values are represented as single dot and the bars represent median. These results suggest that the susceptibility of *T. vaginalis* strains may be influenced by their geographic origin. Statistical significance was tested via the Kruskal-Wallis test.

Anti-*T. vaginalis* activity of the essential oils from *C. citratus, C. grandis*, and *M. arvensis* was tested on the 30 protozoan clinical isolates, and MLC was evaluated after 24 h and 48. As shown in **Figure 3**, *C. citratus* essential oil showed the highest antitrichomonal activity both at 24 h (**Figure 3A**) and 48 h (**Figure 3B**), with the MLC median value of 0.04% and 0.03%, respectively. The median MLC of *M. arvensis* essential oil against 30 *T. vaginalis* isolates was 0.11% (range 0.06 to 0.25%) at 24 h, and 0.075% (range 0.02 to 0.19%) at 48 h. *C. grandis* essential oil showed MLC median values of 0.095% (range 0.02% to 0.4%) at 24 h and median values of 0.06% (range 0.01% to 0.25%) at 48 h. Only for *C. grandis*, the antitrichomonal activity seems to be dependent on the geographic origin of the protozoan. In detail, after 48 h of incubation, the median MLC of Vietnamese isolates was higher than Italian isolates (0.25% and 0.03%, respectively), while no significant differences between Vietnamese and Italian isolates were observed regarding the sensitivity to *C. citratus* and *M. arvensis* essential oils (p-value > 0.99) (**Figure 4**).

**Figure 5:**
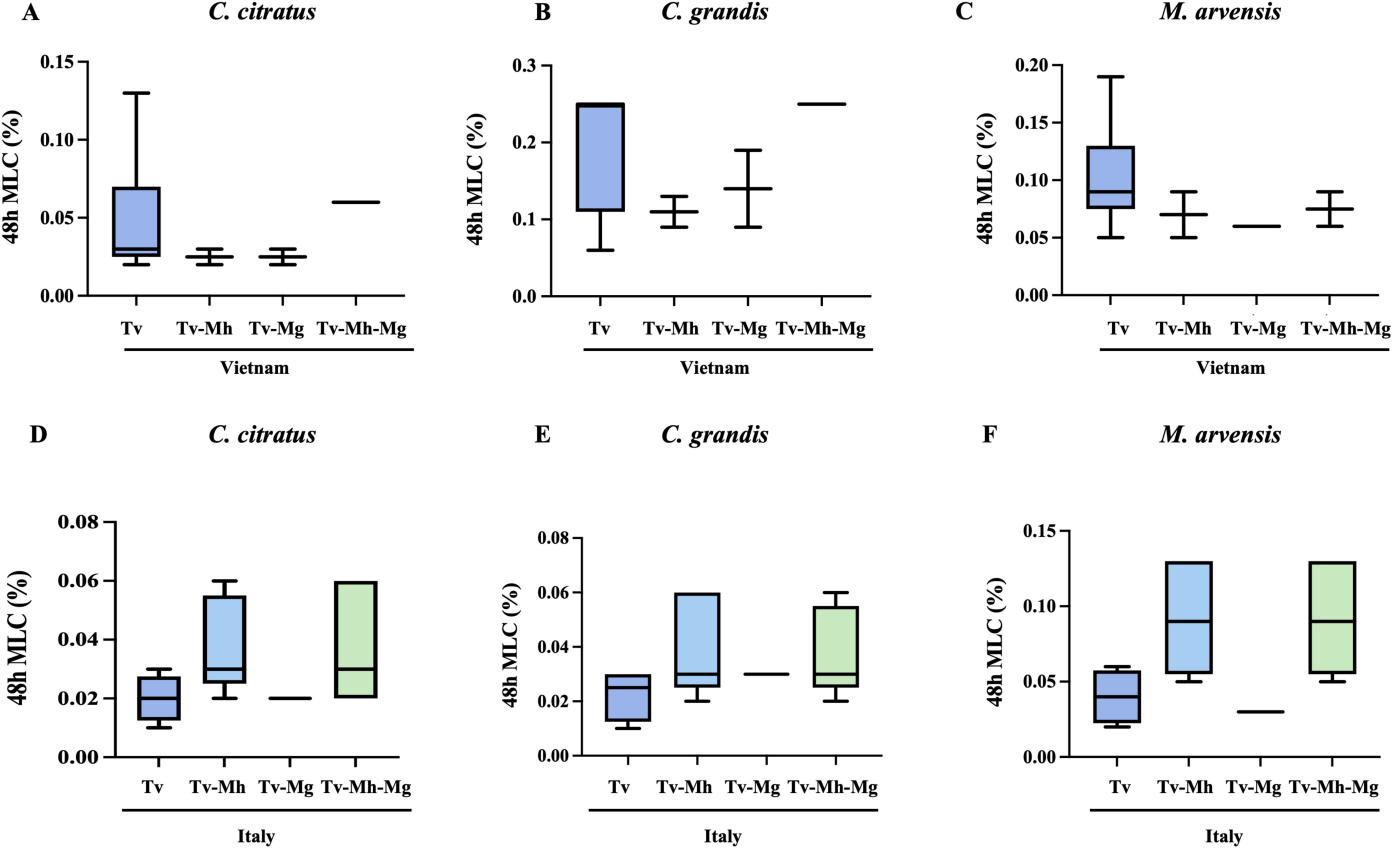
Influence of *M. hominis* and *Ca.* M. girerdii on the susceptibility of Vietnamese **(A–C)** and Italian **(D–F)** *T. vaginalis* strains to essential oils. Tv, *T. vaginalis*; Mh, *M. hominis*; Mg, *Ca.* M. girerdii. The horizontal line within each box indicates the median, while the box boundaries represent the first and third quartiles. The vertical lines (whiskers) extend to the minimum and maximum values obtained from a minimum of 3 samples. Statistical significance was tested via Krustal-Wallis test.

The possibility that the presence of one or both endosymbionts within *T. vaginalis* cells could interfere with sensitivity to essential oils was evaluated, showing no statistically significant difference in the MLC values of *M. hominis* and/or *Ca.* M. girerdii-infected *T. vaginalis* compared with non-infected strains, independently from their geographical origin (**Figure 5** **).**

### 3.5 Evaluation of cytotoxicity of essential oils on HeLa cells

**Figure 6:**
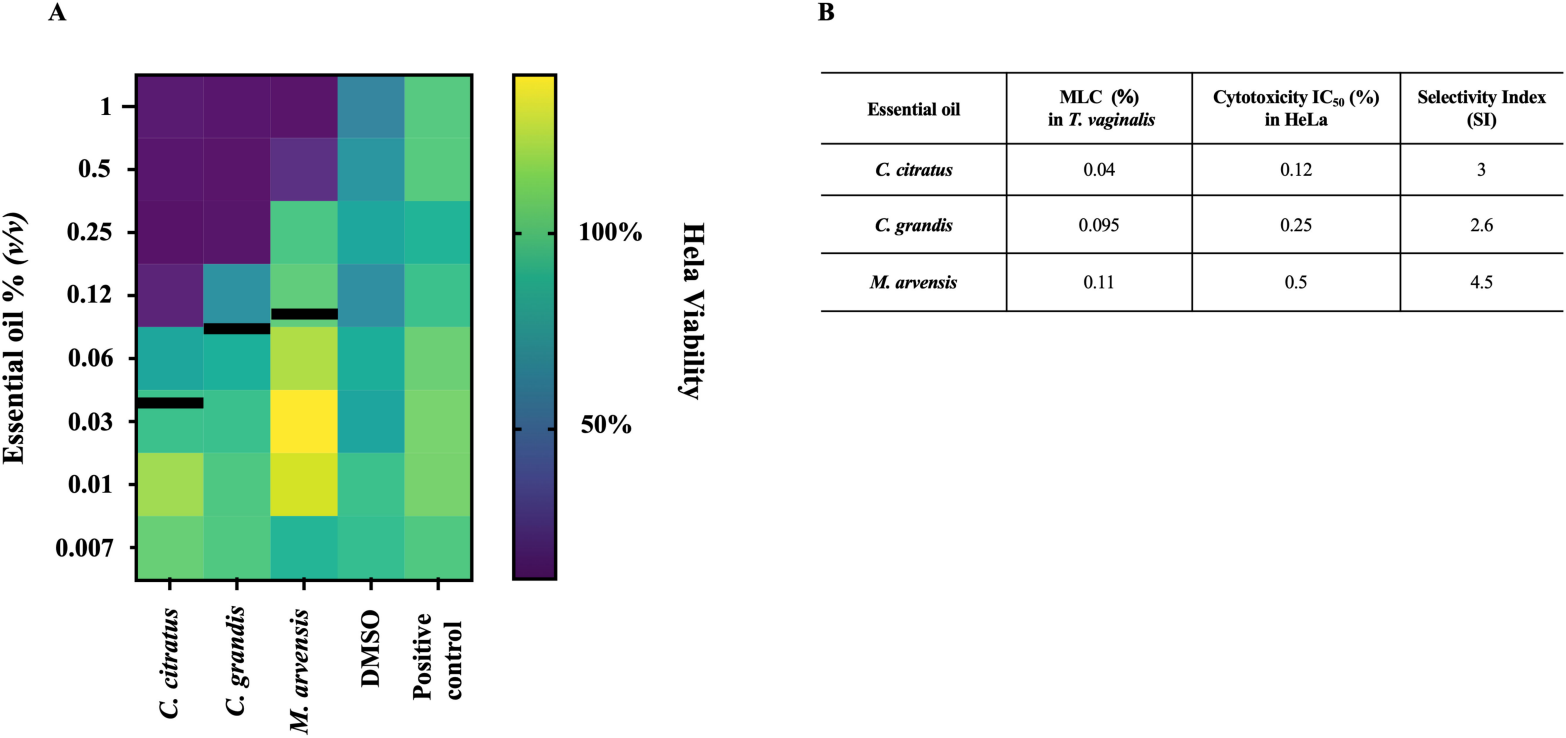
Cytotoxicity effects on HeLa cells. The cytotoxicity effects of three essential oils on HeLa were evaluated through MTT assay after 24h of treatment. **(A)** Heatmap show the percentage of viability of cells at scalar dilution concentration of *C*. *citratus, C. grandis* and *M. arvensis*, showing that all three oils possessed moderate cytotoxicity activity at higher concentrations. Black lines indicate the MLC value at 24 h in *T. vaginalis* strains **(B)** Table shows the MLC value at 24 h in *T. vaginalis* strains compared with IC_50_ value of HeLa cells treated with *C*. *citrus, C. grandis* and *M. arvensis.* SI were determinated by dividing IC_50_ value of Hela cells by the MLC value of *T. vaginalis* strain.

The cytotoxicity of all essential oils was evaluated on HeLa cells after 24h of exposure to concentrations ranging from 1% up to 0.008%, through an MTT assay. Cell viability varies for the different oils tested, with IC_50_ values of HeLa treated with *C. citratus, C. grandis* and *M. arvensis* of 0.12%, 0.25% and 0.5%, respectively (**Figure 6A**). For all essential oil tested, the concentrations able to kill *T. vaginalis* did not exert cytotoxic effects on HeLa cells. Based on the selectivity index, *M. arvensis* EO had the best selectivity index of 4.5, following by *C. citratus* with SI=3 and *C. grandis* with SI= 2.6. (**Figure 6B**).

HeLa cells incubated with medium alone and medium added with scalar concentrations of DMSO were used as controls.

### 3.6 Evaluation of antibacterial effects on *Lactobacillus* species

The minimal inhibitory concentration (MIC) values of *C. citratus*, *C. grandis*, and *M. arvensis* against three clinical isolates of *L. crispatus* and three clinical isolates of *L. gasseri* were evaluated after 24 h of incubation. *C. citratus* and *C. grandis* did not exhibit any inhibitory effects against *Lactobacillus* strains even at the highest concentrations, whereas *M. arvensis* demonstrated a weak effect (MIC of 0.5%) on all strains tested.

## 4 Discussion

Trichomoniasis is associated with severe complications, including increased risks of cervical and prostate cancer (Stark et al., 2009) and adverse pregnancy outcomes, such as preterm delivery, premature rupture of membranes, and low birth weight (Fichorova, 2009). Metronidazole and tinidazole are the only drugs approved for the treatment of trichomoniasis, but a range of side effects, including gastrointestinal disturbances, neurological symptoms, and allergic reactions, can limit patient adherence to the treatment. Furthermore, the emergence of resistance to metronidazole in *T. vaginalis* is an increasing concern that prompts research for alternative or complementary therapeutic options, such as natural compounds, to overcome these limitations and improve treatment outcomes.

In the pharmaceutical industry, natural products are essential for drug development. Approximately 35% of approved drugs are based on natural compounds or their semisynthetic derivatives, and among the 15 antiparasitic drugs approved by health authorities between 1981 and 2006, 65% were either natural products or their derivatives (de Brum Vieira et al., 2015). Among plant derivatives, essential oils have shown to be promising antimicrobial compounds, since a broad spectrum of activities has been demonstrated against bacterial, fungal, and viral infectious agents. However, to data, only a limited number of essential oils have been tested *in vitro* against *T. vaginalis* with variable efficacy (Friedman et al., 2020).

In the present work, we tested for the first time the anti-protozoan activity of essential oils obtained from *C. citratus*, *M. arvensis*, and *C. grandis* against 30 *T. vaginalis* strains isolated from Italian and Vietnamese patients. The decision to use such a high number of strains was driven by the fact that the effect of the oils could be influenced by the phenotypic variability often observed between different *T. vaginalis* isolates, especially when they come from distinct geographical regions (Hirt and Sherrard, 2015). In fact, until now, the anti-trichomonad activity of plant-derived compounds has been tested on a single or only a few *T. vaginalis* strains.

The chemical composition of the three tested essential oils was generally consistent with previously reported literature data. In particular, *C. citratus* essential oil was characterized by a high content of the two isomers, neral and geranial, which together accounted for over 73% of the total composition. Recently, Kiełtyka-Dadasiewicz et al. reviewed the biological and chemical properties of *C. citratus* essential oil, corroborating our overall chemical profile (Kiełtyka-Dadasiewicz et al., 2024). The essential oil from *M. arvensis* has been extensively studied, and its chemical composition is well known to be dominated by menthol (Tiwari, 2016). Our results confirm that the chemical composition of *M. arvensis* essential oil is characterized by a high menthol content, representing over 67% of the total composition, further validating the identity of the source. Moreover, as expected and in accordance with previous literature (Petretto et al., 2022), the chemical composition of *C. grandis* essential oil confirmed the predominance of limonene, which accounted for over 82% of the total composition.

The activity of EOs against *T.vaginalis* isolates was interpreted according to the definition proposed by Pérez Zamora et al. stating that an MLC less than or equal to 0.5 mg/ml (corresponding to a concentration of about 0.06% of essential oil) indicates a strong antimicrobial activity, while values above 1.0 mg/ml (about 0.125%) indicate weak or absent activity (Pérez Zamora et al., 2018). Among the three EOs tested, *C. citratus* exhibited the best anti*-T. vaginalis* activity, with a median MLC value of 0.04% at 24 h and 0.03% at 48 h, while the EO extracted from *M. arvensis* was less efficient, with a median MLC value of 0.11% at 24 h and 0.075% at 48 h. Interestingly, the essential oil of *C. grandis* demonstrated high efficacy against the Italian strains, but was considerably less effective against the Vietnamese isolates, with median MLC values at 48 h of 0.25% and 0.03%, respectively. This significant difference in sensitivity between the two groups of isolates was not observed with the other two oils, nor with metronidazole.

The strong anti-*T. vaginalis* effect of *C. citratus* essential oil is comparable to that described by Ezz Eldin et al. for *Ocimum basilicum*, which, until now, seems to be the most efficient anti-protozoan oil, showing 100% inhibition of growth at a concentration of 30 µg/ml after 24 h of incubation (Ezz Eldin and Badawy, 2015). Other oils, such as *Atalantia sessiflora* and *Leoheo domatiophorus* EOs from Vietnam, have also demonstrated promising effects against *T. vaginalis* (Le et al., 2020). On the other hand, less effective anti-trichomonad activities have been observed for *Foeniculum vulgare*, with MLC values of 0.16% against *T. vaginalis* (Karami et al., 2019), as well as for *Lavandula angustifolia* and *Lavandula intermedia* essential oils (Moon et al., 2006). The excellent anti-*T.vaginalis* activity of *C. citratus* essential oil may be at least partly related to the high levels of neral and geranial, whose antimicrobial activity is associated with their action on the cell membrane (Oladeji et al., 2019).

A peculiar feature of *T.vaginalis* is its ability to establish a stable symbiosis with two different *Mycoplasma* species. From the first description in 1998, the presence of *M. hominis* has been confirmed in *T. vaginalis* isolated worldwide (Fichorova et al., 2017). More recently, a new *Mycoplasma* species, *Ca.* M. girerdii has been described almost exclusively as part of the vaginal microbiota of women infected by *T. vaginalis* (Fettweis et al., 2014), and we recently observed *Ca.* M. girerdii in 61% of clinical *T. vaginalis* isolates from Italy, in the vast majority of cases coexisting with *M.hominis* (Margarita et al., 2022a). The biological association between *T. vaginalis* and mycoplasmas has been shown to significantly impact several aspects of protozoan life and pathogenesis, including drug response (Margarita et al., 2022b, 2016). Our recent results, obtained by comparing the sensitivity to metronidazole of syngenic *T. vaginalis* strains with and without one or both symbionts, suggest that the presence of *M. hominis* increases the sensitivity to metronidazole, while the symbiosis with *Ca.* M. girerdii does not appear to have the same effect (Margarita et al., 2022b).

To test the hypothesis that the presence of one or both symbionts could correlate with the susceptibility of trichomonad isolates to the cytopathic effect of essential oils, we screened all 30 *T. vaginalis* strains by Real-Time PCR for the presence of *M. hominis* and *Ca.* M. girerdii. More than 56% of strains tested positive for the presence of at least one symbiont, with a significant difference between the two geographical groups. In detail, only 40% of Vietnamese strains present at least one mycoplasmas in association with *T. vaginalis* isolated against 73% of Italian strains. The results are consistent with our previous findings, indicating a higher prevalence of *Mycoplasma*-free *T. vaginalis* strains in Vietnam compared to Italy (Fürnkranz et al., 2018; Margarita et al., 2022b). *Ca.* M. girerdii-specific DNA was present in 10 out of 30 *T. vaginalis* isolates analyzed and, among them, seven were co-infected by *M. hominis*. The high rate of simultaneous infection by the two Mycoplasmas in a single trichomonad isolate confirms previous data suggesting that coexistence may represent an advantage for all three symbionts.

No significant differences in susceptibility to the three essential oils were observed between *T. vaginalis* isolates harboring bacterial symbionts and those without, suggesting that the presence of *M. hominis* and *Ca*. M.girerdii does not influence the protozoan’s response to these natural compounds.

In this work, we have also compared the *in vitro* metronidazole sensibility of *T. vaginalis* strains associated and not associated to one or both symbionts, and results were compared to those obtained with EOs. Only one Italian and one Vietnamese isolate showed a reduced MTZ sensitivity. Interestingly, all EOs tested were strongly active against the two *T. vaginalis* isolates showing the weakest sensitivity to MTZ. *Ca.* M. girerdii presence seems to not influence the sensitivity to MTZ of *T. vaginalis* strains, while the presence of *M. hominis* seems not to be correlated with higher sensitivity in the protist, in contrast with our previous study. These data confirm the high variability among *T. vaginalis* strains. Interestingly, the only two strains showing a reduced sensitivity to MTZ (MLC value > 17 µg/ml) are totally Mycoplasma-free, confirming our previous data (Margarita et al., 2022b).

None of the three tested essential oils, at the concentrations effective on the protozoon, alter the viability of treated HeLa cells. In particular *C. citratus*, the best performing anti-*T.vaginalis* EO is effective at a concentration of 0.04% that is three-fold lower than its IC_50_ evaluated on HeLa cells. According to ISO 10993-5, a treatment concentration is considered non-cytotoxic if the *in vitro* viability of cells tested is above 80%, within 80%–60% weak; 60%–40% moderate and below 40% strong cytotoxicity respectively (International Organization for Standardization; Geneva, 2009).

An ideal anti-*T. vaginalis* drug should not affect the vaginal microbiota and in particular lactobacilli, due to their protective role in maintaining a health vaginal environment. *L. crispatus* and *L. gasseri* play a key role in maintaining vaginal health by contributing to the dominance of a healthy vaginal microbiota. The presence of *T. vaginalis* is linked to a decrease in lactobacilli and an increase in the number of anaerobic bacteria associated with bacterial vaginosis (Brotman et al., 2010). Several studies have demonstrated that the interaction of *T. vaginalis* with dysbiotic vaginal microbiota species modulates the host’s inflammatory response, contributing to pathogenesis (Fichorova et al., 2013; Hinderfeld et al., 2019; Hinderfeld and Simoes-Barbosa, 2020). *L. crispatus* and *L. gasseri* play a key role in maintaining vaginal health by contributing to the dominance of a healthy vaginal microbiota. *L. crispatus* contribute to maintaining vaginal health by preserving an acidic environment, preventing the overgrowth of pathogenic microorganisms, and supporting the immune system’s defense mechanisms. On the other hand, Phukan et al. demonstrated that *L. gasseri* can inhibit the adhesion of *T. vaginalis* to human vaginal cells (Phukan et al., 2013), thereby exerting a protective effect by reducing the cytopathic activity of the protozoan (Fiori et al., 1999). We evaluated the antibacterial activity of the three EOs on different strains of *L. crispatus* and *L. gasseri*, demonstrating that *C. citratus* and *C. grandis* do not exhibit any inhibitory effects against *Lactobacillus* strains even at the highest concentrations. *M. arvensis* essential oil was the only one to exhibit a slight effect against *Lactobacillus* species. However, at the concentrations effective against *T. vaginalis*, it did not display any bactericidal activity toward lactobacilli. The finding that *C. citratus* essential oil, which exhibited the strongest anti-*T. vaginalis* activity, is completely ineffective against the two protective *Lactobacillus* species is particularly noteworthy. This is especially relevant considering that a significant antimicrobial effect of this EO had previously been reported against various bacterial species (Kiełtyka-Dadasiewicz et al., 2024).

To assess the potential toxicity against human cells, we tested the effect of the EOs on HeLa cells showing that, at concentrations effective against *T. vaginalis*, the essential oils do not exhibit significant cytotoxic effects on HeLa. In fact, the concentrations that inhibit the parasite are lower than those that affect cell viability showing a high Safety Index for all EOs tested (**Figure 6B**). Although only one cell line was evaluated, the results obtained suggest a favorable safety profile for the potential use of these oils in therapeutic treatments.

In conclusion, the essential oil of *C. citratus* demonstrate the best anti-*T. vaginalis* activity, while exhibiting no cytotoxic effects at the concentrations effective against the parasite. These findings highlight the potential of essential oils as alternative or complementary therapeutic options for trichomoniasis, with a favorable safety profile for human cells. Further studies exploring the potential synergistic effects of *C. citratus* essential oil in combination with MTZ could offer valuable insights, especially in the context of drug-resistant isolates. The lack of inhibitory effects on beneficial *Lactobacillus* species further supports their suitability for preserving vaginal health, making these oils promising candidates for the development of safer, more effective treatments for trichomoniasis and associated complications.

## Data Availability

The original contributions presented in the study are included in the article/**Supplementary Material**. Further inquiries can be directed to the corresponding author.
