# Supplementary material for: Effect of essential oils from Cymbopogon citratus, Citrus grandis, and Mentha arvensis on Trichomonas vaginalis and role of its symbionts Mycoplasma hominis and Ca. Mycoplasma girerdii
Source: Front Parasitol. 2025 Aug 14;4:1610965. doi: 10.3389/fpara.2025.1610965 (PMC12392280; doi:10.3389/fpara.2025.1610965)
Supplement: Supplementary file 1 [file Table1.docx]

**Supplementary Table S1**. **Main compounds detected in the *Cymbopogus citratus* essential oil.** Results were expressed as relative percentage obtained by peak area normalization (A%). RI_(WAX)_ retention index calculated on an ZB-WAX column, RI_(lit)_: literature retention index on WAX columns from those reported in the NIST Chemistry WebBook database.

| ***C. citratus*** | **A%** | **RI** | **Lit** |
| --- | --- | --- | --- |
| β-Myrcene | 4.2 | 1161 | 1162 |
| 2,3-Dehydro-1,8-cineole | 0.4 | 1182 | 1195 |
| Limonene | 0.6 | 1191 | 1196 |
| Eucalyptol | 0.2 | 1197 | 1198 |
| β-Ocimene (cis) | 0.6 | 1232 | 1231 |
| β-Ocimene (trans) | 0.5 | 1248 | 1233 |
| 5-Hepten-2-one, 6-methyl- | 2.2 | 1334 | 1333 |
| p-Menthan-3-one | 0.2 | 1450 |  |
| β-Pinene oxide | 0.1 | 1471 |  |
| Citronellal | 0.2 | 1474 | 1474 |
| Linalool | 2.0 | 1548 | 1549 |
| cis-Verbenol | 0.5 | 1570 | 1629 |
| α-Bergamotene | 0.4 | 1576 | 1573 |
| β-Caryophyllene | 0.4 | 1581 | 1584 |
| 1-Menthol | 0.9 | 1630 | 1639 |
| Neral | 30.3 | 1676 | 1667 |
| Geranyl formate | 0.4 | 1703 | 1709 |
| Geranial | 43.3 | 1738 | 1744 |
| Geranyl acetate | 3.6 | 1761 | 1750 |
| Citronellol | 0.6 | 1771 | 1781 |
| cis-Geraniol | 0.4 | 1798 | 1796 |
| Isogeraniol | 0.1 | 1808 | 1820 |
| trans-Geraniol | 6.2 | 1851 | 1842 |
| Caryophyllene oxide | 0.2 | 1970 | 1988 |
| 5-(1-Hydroxy-1-methylethyl)-2-methyl-2-cyclohexen-1-one | 0.2 | 2040 |  |
| Cedrol | 0.0 | 2060 | 2065 |
| 2H-1-Benzopyran, 7-methoxy-2,2-dimethyl- | 0.2 | 2077 | 2068 |
| Selina-6-en-4-ol | 0.2 | 2157 |  |
| Cembrene | 0.2 | 2202 | 2207 |
| Geranic acid | 0.7 | 2354 | 2353 |

**Supplementary Table S2**. **Main compounds detected in the *Citrus grandis* essential oil.** Results were expressed as relative percentage obtained by peak area normalization (A%). RI_(WAX)_ retention index calculated on an ZB-WAX column, RI_(lit)_: literature retention index on WAX columns from those reported in the NIST Chemistry WebBook database.

| ***C. citrus*** | **A%** | **RI** | **RI lit** |
| --- | --- | --- | --- |
| α-Pinene | 0.6 | 1041 | 1039 |
| β-Pinene | 0.7 | 1102 | 1108 |
| Sabinene | 0.1 | 1115 | 1109 |
| β-Myrcene | 1.3 | 1161 | 1162 |
| Limonene | 82.3 | 1196 | 1196 |
| eucalyptol | 0.2 | 1203 | 1198 |
| p-cymene | 1.7 | 1264 | 1266 |
| Limonene oxide, (not identified isomer) | 1.4 | 1435 | 1440 |
| Limonene oxide, (not identified isomer) | 1.0 | 1448 | 1440 |
| trans-p-Mentha-2,8-dienol | 0.4 | 1540 |  |
| 4-Acetyl-1-methylcyclohexene | 0.5 | 1547 | 1568 |
| cis-Verbenol | 1.0 | 1620 | 1629 |
| -(-)-Menthol | 0.2 | 1630 | 1642 |
| 2-Cyclohexen-1-ol, 1-methyl-4-(1-methylethenyl)-, trans- | 0.9 | 1661 | 1670 |
| Neral | 0.2 | 1678 | 1667 |
| α-Terpineol | 0.2 | 1689 | 1692 |
| Carvone | 2.0 | 1725 | 1722 |
| Geranial | 0.4 | 1753 | 1744 |
| (-)-Perillaldehyde | 0.1 | 1774 | 1776 |
| p-Mentha-1(7),8-dien-2-ol | 0.1 | 1794 | 1797 |
| p-Mentha-1,8-dien-3-one | 0.3 | 1829 | 1833 |
| Carveol (not identified isomer) | 1.5 | 1835 | 1877 |
| Carveol (not identified isomer) | 1.0 | 1866 | 1877 |
| p-Mentha-1(7),8(10)-dien-9-ol | 0.1 | 1996 |  |
| p-Mentha-1,8-dien-7-ol | 0.1 | 2003 | 2005 |

**Supplementary Table S3**. **Main compounds detected in the *Mentha arvenis* essential oil.** Results were expressed as relative percentage obtained by peak area normalization (A%). RI_(WAX)_ retention index calculated on an ZB-WAX column, RI_(lit)_: literature retention index on WAX columns from those reported in the NIST Chemistry WebBook database.

| ***M. arvensis*** | **A%** | **RI** | **RI_lit** |
| --- | --- | --- | --- |
| Limonene | 0.2 | 1191 | 1196 |
| Eucalyptol | 0.1 | 1197 | 1198 |
| Menthone | 14.4 | 1455 | 1448 |
| Isomenthone | 5.7 | 1480 | 1465 |
| β-Bourbonene | 0.3 | 1491 | 1500 |
| Menthyl acetate | 0.1 | 1521 | 1547 |
| Isomenthol acetate | 2.2 | 1556 | 1547 |
| Cyclohexanol, 1-methyl-4-(1-methylethyl)- | 0.4 | 1560 | 1621 |
| Caryophyllene | 0.5 | 1582 | 1584 |
| Neo-Menthol | 4.6 | 1594 | 1574 |
| Neoisomenthol | 0.2 | 1620 | 1605 |
| Menthol | 67.2 | 1640 | 1639 |
| Isomenthol | 0.1 | 1643 | 1667 |
| isomenthol | 2.7 | 1660 | 1684 |
| Piperitone | 0.1 | 1715 | 1714 |
| 1-Decanol | 0.4 | 1767 | 1763 |
| Thymol | 0.3 | 2194 | 2189 |
